# It's all in the details: A call for administering the COVID-19 vaccine in Lebanon through a transparent and un-politicized collaborative approach

**DOI:** 10.1016/j.eclinm.2021.100748

**Published:** 2021-02-05

**Authors:** Shadi Saleh, Hady Naal, Ali H. Mokdad

**Affiliations:** a Global Health Institute, American University of Beirut, Beirut, Lebanon; b Faculty of Health Sciences, American University of Beirut, Beirut, Lebanon; c Institute for Health Metrics and Evaluation, University of Washington, Seattle, WA, United States

Lebanon is a small low-to middle-income country located in the Eastern Mediterranean whose history is rife with conflicts and political corruption that compromised, and are currently threatening, its stability and development [1]. Two recent events significantly exposed decades of inherent corruption and its dysfunctional system: a severe economic crisis that is arguably the worst since the 1930s Great Depression, and the tragic Beirut Port explosion which resulted in hundreds of thousands of damaged homes, thousands of injuries, and hundreds of deaths [2]. While struggling to cope with these crises and while operating under an overstretched healthcare system, Lebanon is additionally burdened with the spread of COVID-19. Similar to many other countries, Lebanon has been facing exponential surges of cases in recent months [3]. However, this has been exacerbated by inadequate handling of the pandemic response at different levels, including poor lockdown decisions and enforcement, inefficient health system responses, limited facilities upgrade to manage expected hospitalization, and poor governmental coordination among others [2,4]. In mid-January 2021, the Lebanese Government finalized the regulatory requirements to import vaccines, albeit in limited amounts, after unproductive debates on waiving liabilities associated with potential adverse events [5]. The major challenge in 2021 for Lebanon, along with the economic and stability challenges, is to ensure a fair, clientelism-free, and transparent allocation of vaccines.

At the time of preparing this commentary, Lebanon remains in a state of emergency, and in a race against time, given the rate at which COVID-19 is mutating and causing serious damages [6]. Despite that, the national governmental response has been inadequate, as demonstrated by increases in infections and death rates, and by lack of transparency with regards to its response strategy during the past year. This has reverberated across the healthcare system, causing disruptions such as long delays from intensive care unit preparations to triage, and limited equity in treatment and access to services [4]. Hospitals have expressed inability to handle cases, leading to complementary patient management methods such as coordinating home care and using alternative locations [7]. Without a clearly communicated strategy describing how COVID-19 will be managed, and with the country's failure to control infections, administering vaccines is the only way out of the pandemic. In such a climate of poor implementation and political corruption, a major concern is that this may be used for political gains at the expense of the population's health. Although a general vaccination strategy roadmap, generated based on good global practices, has been shared [8,9], it lacks crucial details that may lead to misuse, specifically with the absence of concrete plan for logistics of administration. This is problematic considering that the challenge in Lebanon has always been with the implementation of policies. In this case, how can the policy's implementation be equitable and isolated from political interference that would result in clientelism and inefficiencies, leaving the most in need for the vaccine unable to access it.

The vaccines should be received in a centralized manner for efficient storage, management, and distribution, while ensuring no vaccine wastages once a vile is opened. Although coastal areas are most populated, delivering the vaccine to remote locations is critical to reduce transportation burdens. With limited resources, alternative medical set-ups such as schools and municipalities, present important opportunities to administer the vaccine, since are typically used to host public activities. For instance, during the elections, official lists of individuals from given catchment areas are pre-issued, and subsequently people are given permission to vote in centralized locations. Seeing its effectiveness, similar methodologies can be implemented for vaccine administration, whereby pre-issued lists are segregated by age and then invited in set times, while preparing backup plans to call qualified individuals. In parallel, because it is pivotal to have general population buy-in, communication and advocacy campaigns should (a) convince sceptical individuals and motivate them to take the vaccine, and (b) improve health literacy regarding COVID-19 and address misconceptions. Ideally, this initiative may warrant collaborations with international partners such as the World Health Organization or the United Nations.

Special consideration should be given to refugees, representing around 25% of the population, along with others such as domestic workers and inmates. It is important that all residents in Lebanon equitably access the vaccine to stop the spread of COVID-19 nationally and beyond.

Although primarily a global health issue, collaborative action to coordinate the COVID-19 vaccination initiative may reverberate across wider sectors and initiate momentum for future efforts that send a clear message that there is still hope somewhere for the country to recover independently of political corruption, clientelism, and inefficiency.

## Declaration of Competing Interest

Authors declare no conflict of interest.
